# *Bacillus* from the Brazilian Caatinga biome enhances maize productivity and agronomic performance under rainfed conditions across multi-location field trials

**DOI:** 10.3389/fpls.2026.1867580

**Published:** 2026-07-15

**Authors:** Ubiraci Gomes de Paula Lana, Sylvia Morais de Sousa, Bárbara Temponi Vilarino Godinho, Lourenço Vitor Silva Ferreira, Christiane Abreu de Oliveira Paiva, João Vitor Silvério Alves de Avelar, Paulo César Magalhães, Julio Cezar Souza Vasconcelos, Geraldo Magela de Almeida Cançado, Eliane Aparecida Gomes

**Affiliations:** 1 Embrapa Maize and Sorghum, Sete Lagoas, Minas Gerais, Brazil; 2 Federal University of Lavras, Lavras, Minas Gerais, Brazil; 3 Federal University of São João Del-Rei, São João Del-Rei, Minas Gerais, Brazil; 4 Embrapa Digital Agriculture, Campinas, São Paulo, Brazil

**Keywords:** bioinoculants, brazilian semi-arid, drought tolerance, rainfed agriculture, water deficit, *Zea mays* L.

## Abstract

Drought stress is among the most critical limitations to maize productivity, particularly under rainfed conditions. In this study, we explored the Brazilian Caatinga biome as a source of drought adapted plant growth-promoting bacteria and evaluated their potential to mitigate drought effects in maize (*Zea mays* L.). A total of 414 thermo-tolerant bacterial strains were isolated from soil, of which 28 *Bacillus* strains were able to grow under low water activity. These strains exhibited multiple plant growth-promoting traits *in vitro*, including exopolysaccharide production, biofilm formation, siderophore production, indole-3-acetic acid synthesis, putative nitrogen fixation, and phosphate solubilization. Twelve selected strains significantly improved root morphology, relative chlorophyll content (SPAD units), and biomass accumulation in maize seedlings under osmotic stress induced by polyethylene glycol. Notably, strain 1A11 showed the most consistent effects, promoting root growth and biomass accumulation under both stressed and non-stressed conditions, indicating constitutive growth promotion across environments, whereas other strains showed stronger responses under stress. This stability across environments strengthens its agronomic value, particularly in regions characterized by high rainfall variability. Genome sequencing of five elite strains (1A11, 5D5, 6E9, 1H10, and 2E7) identified conserved gene clusters associated with exopolysaccharide production, indole-3-acetic acid synthesis, phosphate metabolism, iron acquisition (siderophore synthesis), synthesis of volatile compounds, motility, chemotaxis, and general responses to osmotic and oxidative stress. Multi-location field trials conducted across five locations in Brazil, under rainfed conditions, indicate that strains 1A11 (*Bacillus subtilis*), 5D5, and 6E9 (*Bacillus velezensis*) consistently increased grain yield compared to the non-inoculated control and performed similarly to or better than a commercial inoculant. Mean productivity gains with the strain 1A11 reached up to 39% relative to the non-inoculated treatment across environments. These results indicate that *Bacillus* strains isolated from semi-arid soils were able to convert multifunctional potential into measurable agronomic gains under field conditions, demonstrating their potential as bioinoculants to enhance maize resilience under water-limited agricultural systems.

## 1 Introduction

Drought stress is among the most critical constraints on modern agriculture, significantly reducing crop yields and threatening global food security (Naumann et al., 2018; Marchin et al., 2020). Prolonged dry periods, particularly in rainfed systems across Africa, Latin America, and South Asia, have been linked to yield losses of up to 40% in staple crops such as maize, wheat, and rice (Zampieri et al., 2023). Projections indicate that by 2050, drought could reduce the arable land available by 40-50%, underscoring the urgent need for climate-resilient agricultural strategies (Kraklow et al., 2025).

In Brazil, particularly in the Central-West region, the country’s main grain-producing area, prolonged dry periods occurring in the middle of the crop cycle, commonly referred to as “dry spells” or “veranicos”, are frequent (Assad et al., 1993). These events reduce water availability during critical phenological stages of maize and soybean, increasing the risk of yield losses, given the region’s high susceptibility to prolonged dry spells. Long-term assessments indicate average drought-related yield losses above 10%, reaching up to 40–60% under severe water stress conditions (Ferreira et al., 2024).

Drought disrupts multiple physiological and biochemical processes in plants, including reduced photosynthetic efficiency and transpiration rate, impaired nutrient uptake, hormonal imbalances, flower abortion before seed set, and overproduction of reactive oxygen species, culminating in cellular and molecular damage that severely limits productivity (Selvakumar et al., 2012; Sies and Jones, 2020; Kumar et al., 2024). As the frequency and intensity of drought events increase due to climate change, there is a growing emphasis on identifying sustainable, nature-based approaches to enhance crop resilience (Gebrechorkos et al., 2025). In this context, plant growth-promoting bacteria (PGPB) are promising biotechnological tools for mitigating biotic and abiotic stress, representing a sustainable alternative to conventional agricultural inputs and contributing to global food security in line with the United Nations Sustainable Development Goals (SDGs) (Tsotetsi et al., 2022; Cao and Solangi, 2023; Toukabri and Mohamed Youssef, 2023).

These beneficial microbes mitigate the effects of drought through a variety of mechanisms. The secretion of exopolysaccharides (EPS) by some strains promotes biofilm formation and root adherence, maintaining a hydrated microenvironment around the rhizosphere that helps buffer plants against desiccation, salinity, and thermal stress (Morcillo and Manzanera, 2021). PGPB can also activate plant antioxidant defense systems (e.g., superoxide dismutase, catalase) and promote osmolyte accumulation (e.g., proline, soluble sugars), minimizing oxidative damage and stabilizing osmotic potential during drought episodes. In addition, PGPB employ a variety of adaptation strategies to boost plant resilience, including synthesis of phytohormones (e.g., auxins, gibberellins, cytokinins), production of 1-aminocyclopropane-1-carboxylate (ACC) deaminase, an enzyme that lowers ethylene levels, regulation of nitrogen fixation, and phosphate solubilization (Kavamura et al., 2013; Vieira Velloso et al., 2020).

Recent advances in genomics and transcriptomics have now revealed the genetic basis of these adaptive traits. Microbes from arid ecosystems often harbor genes involved in osmotic balance, reactive oxygen species detoxification, and ion transport, which are markers of their resilience and potential utility as bioinoculants (Mousa et al., 2024). These genomic results enable the identification and selection of elite strains for agricultural application, with potential enhancement through gene editing or functional validation.

In this context, the present study focused on the Brazilian semi-arid Caatinga, a savanna-like biome, and its soils as reservoirs of bacterial diversity, aiming to identify *Bacillus* strains with potential to enhance plant performance under water-limiting conditions. Specifically, the study addressed three main objectives: (i) isolation and characterization of *Bacillus* strains using physiological and functional traits associated with tolerance to dehydration/osmotic stress and plant growth promotion; (ii) genome sequencing and annotation of selected strains to identify genetic determinants potentially related to stress resilience; and (iii) field evaluation of maize yield following inoculation with selected *Bacillus* strains under agronomically relevant conditions.

We hypothesize that *Bacillus* strains isolated from the Caatinga soil harbor adaptive genomic traits that confer enhanced resilience to water-limited conditions and translate into improved maize productivity under field conditions. Collectively, these experiments were key to developing and validating a PGPB-based inoculant that enhances maize yield in water-limited agricultural systems in Brazil.

## 2 Materials and methods

### 2.1 Isolation and selection of bacterial strains

To isolate bacterial strains, soil samples (0–20 cm depth) were collected from different locations in the Caatinga biome in the State of Ceará, Brazil. A 1.0 g aliquot of each soil sample was transferred to a 15 mL conical tube containing 5 mL of 0.85% (w/v) NaCl solution and agitated for 24 h. After homogenization, a heat shock treatment (65 °C for 30 min, then on ice for 5 min) was applied to selectively enrich for spore-forming bacteria of the genus *Bacillus*. Aliquots (50 *µ*L) of each bacterial suspension were plated on tryptic soy agar (TSA; g L^−1^: casein peptone 15, soy peptone 5, NaCl 5, agar 15), prepared at 10% (w/v) and incubated at 40 °C for 72 h. The strains were cryopreserved in a glycerol-based medium and subsequently grown in TSA medium supplemented with sorbitol at concentrations of 405, 520, and 780 g L^−1^, corresponding to water activity (*a_w_*) values of 0.919, 0.897, and 0.807, respectively. Isolates capable of growing on media containing 520 and/or 780 g L^−1^ sorbitol were selected for subsequent molecular identification.

### 2.2 16S rDNA identification

Bacterial genomic DNA was extracted with the Wizard Genomic DNA Purification Kit (Promega, USA) and amplified with 16S rDNA primers 8F and 1492R (Turner et al., 1999). PCR reactions were performed with 30 ng of bacterial genomic DNA, 2.5 *µ*L 10X PCR buffer (20 mM Tris–HCl, pH 8.4, 50 mM KCl), 0.4 *µ*M of each primer, 100 *µ*M dNTPs, 2.5 mM MgCl_2_, and 1 U Taq DNA polymerase (Invitrogen, USA) in a total volume of 25 *µ*L. PCR was performed under the following conditions: 2 min at 95 °C, followed by 35 cycles of 30 s at 94 °C, 30 s at 55 °C, and 2 min at 72 °C. Finally, reactions were incubated for 10 min at 72 °C.

The amplification products were purified with the ExoSAP-IT Kit (Affymetrix, USA) and sequenced with primers 8F, 1492R, 515F (Turner et al., 1999), and 902R (Hodkinson and Lutzoni, 2009) using the Big Dye Terminator v3.1 kit as recommended by the manufacturer (Applied Biosystems, USA). The samples were analyzed using the ABI PRISM 3500XL Genetic Analyzer (Applied Biosystems, USA), and DNA sequences were compared using the BLASTN program (Altschul et al., 1997).

### 2.3 Plant-growth-promoting mechanisms *in vitro*

#### 2.3.1 Exopolysaccharides production

The capacity of bacterial exopolysaccharide (EPS) production was assessed following the method described by Guimarães et al. (1999). Filter paper discs, 5 mm in diameter (Whatman 42), were placed in Petri dishes containing the culture medium and inoculated with 5 *µ*L of each bacterial isolate culture grown in TSB. The plates were incubated at 30 °C for 24 h, and EPS production was assessed by the absence or presence of a mucous colony around the discs. EPS production was confirmed by mixing a platinum loop impregnated with the colony in 2 mL of absolute ethanol; a positive result produced a precipitate, and a negative result showed turbidity.

#### 2.3.2 Biofilm formation

The biofilm-forming capacity was evaluated using the method described by Kasim et al. (2016), with modifications. Initially, 2 *µ*L of bacterial culture (10^8^ cells mL^−1^; OD_540nm_ = 1.0) were inoculated into 200 *µ*L of TSB supplemented with 1% (w/v) glucose in 96-well polystyrene microplates. The assays were performed in triplicate, including a blank well containing only the culture medium. The plates were incubated at 30 °C for 48 h.

After incubation, the medium was removed by inverting the plate, and each well was washed with 200 *µ*L of deionized water. The plates were then inverted onto absorbent paper to dry. Subsequently, 200 *µ*L of methanol was added to each well to fix any biofilm present, and the mixture was incubated at room temperature for 20 min. The methanol was then discarded, and the plates were air-dried. After drying, 200 *µ*L of 0.5% (w/v) crystal violet solution was added to each well, and the plates were incubated for 15 min. The stain was removed by inversion, and the wells were rinsed with deionized water and left to dry at room temperature. Finally, 200 *µ*L of absolute ethanol was added, and the plates were incubated for 30 min.

Absorbance was measured at OD_570nm_ using a UV/VIS spectrophotometer (FLUOstar Omega, BMG LABTECH, Germany). Bacterial strains that retained crystal violet were classified as biofilm producers.

#### 2.3.3 Siderophore production

Siderophore production was assessed by inoculating bacterial strains in triplicate onto nutrient agar plates and incubating at 37 °C for 16 h. A thin overlay of Chrome Azurol S (CAS) medium, as described by Schwyn and Neilands (1987), was then added to each plate. The plates were further incubated at 25 °C for four days. Strains inducing a visible color change in the medium were considered siderophore producers.

#### 2.3.4 Biological nitrogen fixation

The atmospheric nitrogen fixation potential of the bacterial strains was evaluated in triplicate using nitrogen-free semi-solid NFb medium, as described by Döbereiner (1995). Tubes containing 3 mL of NFb medium were inoculated with 10 *µ*L of bacterial culture (10^8^ cells mL^−1^; OD_540nm_ = 1.0) and incubated at 30 °C for 10 days. Positive nitrogen-fixing activity was indicated by the presence of a visible subsurface pellicle in the medium.

#### 2.3.5 Production of acid and alkaline phosphatases

Bacterial strains were first grown in TSB medium for 48 h. Then, a 500 *µ*L aliquot of the culture (10^8^ cells mL^−1^; OD_540nm_ = 1.0) was transferred to 50 mL of NBRIP medium (Nautiyal, 1999) in a 125 mL Erlenmeyer flask, in triplicate. Cultures were incubated at 30 °C and 150 rpm for 72 h. After incubation, the cultures were transferred to 50 mL conical tubes and centrifuged at 8,000 x g for 10 min. The supernatant was filtered through Whatman No. 42 paper, and 150 *µ*L of the filtrate was transferred to 2.0 mL microcentrifuge tubes. Samples were then processed following the protocol described by Tabatabai (1994).

A standard curve was prepared using a *p*-nitrophenol solution at 10 *µ*g mL^−1^. Colorimetric reactions were measured at 400 nm using a LAMBDA Bio spectrophotometer (Perkin Elmer, Germany).

#### 2.3.6 Indole-3-Acetic Acid-like production

The production of tryptophan-dependent IAA-like molecules was measured using the colorimetric method described by Patten and Glick (1996). Each strain was grown in 50 mL of liquid TSB culture medium supplemented with 1.0 mg mL^−1^ tryptophan and incubated at 30 °C for five days at 100 rpm in the dark. After centrifugation for 10 min at 4,000 × g, 0.1 mL of the supernatant was mixed with 0.1 mL of Salkowski reagent (Loper and Schroth, 1986) and incubated in the dark for 20 min.

IAA-like molecule concentration was estimated by measuring absorbance at 540 nm using a UV/VIS spectrophotometer (FLUOstar Omega, BMG LABTECH, Germany). All experiments were performed in triplicate. The development of a pink color indicated a positive result. IAA concentration in the culture medium was determined by comparison with a standard curve generated using commercial IAA at concentrations of 0, 10, 20, 40, 80, and 100 *µ*g mL^−1^.

#### 2.3.7 Phosphate solubilization

All isolates were inoculated into NBRIP growth medium, as described by Nautiyal (1999) with modifications. A standardized bacterial culture (10^8^ cells mL^−1^; OD_540nm_ = 1.0) was inoculated into 50 mL of NBRIP medium and incubated at 30 °C and 150 rpm for nine days. An uninoculated medium served as the negative control.

To quantify soluble phosphorus, the cultures were centrifuged at 10,000 × g for 5 min, and the supernatant was filtered through Whatman No. 42 paper. A 500 *µ*L aliquot of the filtrate was transferred to a new tube, mixed with 5 mL of deionized water and 1 mL of reagent mixture (sulfuric acid, ammonium molybdate, potassium antimony tartrate, and ascorbic acid), and subsequently used for phosphorus determination by the molybdenum blue method according to Murphy and Riley (1962). After 20 min of incubation, soluble phosphorus was quantified by colorimetry at 880 nm using a LAMBDA Bio spectrophotometer (Perkin Elmer, Germany).

A standard curve was prepared using KH_2_PO_4_ solutions at phosphorus concentrations ranging from 0 to 14 mg L^−1^, and the corresponding regression equation was calculated.

### 2.4 Maize inoculation with *Bacillus* strains under osmotic stress in hydroponic conditions

Based on *in vitro* results, 12 *Bacillus* strains were selected and grown separately in Tryptic Soy Broth (TSB) at 30 °C and 120 rpm for three days. After the incubation period, cultures were centrifuged at 4,000 × g for 10 min. Bacterial suspensions were adjusted to an absorbance equal to or higher than 1 at a wavelength of 540 nm to obtain a concentration of 10^8^ colony forming units (CFU) mL^−1^ in 0.85% (w/v) NaCl solution.

Seeds of the maize genotype L521236/CMSM036 (Embrapa Maize and Sorghum) were inoculated with 200 *µ*L of each bacterial suspension per 100 seeds, along with 0.03 g of Bioma Fix^®^. After air drying at room temperature, the seeds were placed in germination paper rolls moistened with deionized water. Uninoculated plants were treated with 0.85% (w/v) NaCl solution and included, along with two experimental control strains, B119 (*Priestia megaterium*) and B2084 (*Bacillus subtilis*), obtained from the Embrapa Collection of Multifunctional and Phytopathogenic Microorganisms of Maize and Sorghum.

After three days of germination, uniform seedlings were transferred to trays containing 8 L of half strength Hoagland’s nutrient solution, pH 5.65 (Liu et al., 1998), and grown for seven days. The nutrient solution was changed every three days. The seedlings were then maintained in the nutrient solution under constant aeration at controlled day/night temperatures (27/20 °C), a 12 h photoperiod, and a light intensity of 330 *µ*mol photons m^−2^ s^−1^ for 10 days.

The seedlings were subsequently transferred to half-strength Hoagland’s nutrient solution supplemented with 10% (w/v) polyethylene glycol 6000 (PEG) (osmotic potential of −0.15 MPa) and maintained under these conditions for three days to induce osmotic stress.

Subsequently, relative chlorophyll content was measured in the upper third of the leaf using a portable SPAD-502 chlorophyll meter (KONICA MINOLTA, Japan), in triplicate. Roots were separated from the shoot, scanned, and analyzed using WinRhizo v. 4.0 (Regent Systems, Quebec, Canada). The root traits analyzed were total root length, total root surface area, surface area of roots with diameters between 0 and 1 mm, 1 and 2 mm, and *>* 2 mm (cm^2^), shoot, root, and total dry weight (g) (Morais de Sousa et al., 2012).

### 2.5 Genome sequencing

Based on the results of the *in vitro* and hydroponic assays, five *Bacillus* strains (6E9, 5D5, 1H10, 2E7, and 1A11) were selected for genome sequencing. The samples were sequenced and analyzed as described by Silva Ferreira et al. (2025). Genome annotation was carried out using PROKKA version 1.8 and RAST version 2.0 (Rapid Annotation using Subsystem Technology) (Seemann, 2014; Aziz et al., 2008; Overbeek et al., 2014).

Functional annotation was performed using the software Protein ANNotation with Z-score – PANNZER (Koskinen et al., 2015), and manual annotation via BlastP of growth-promoting genes reported in the literature. Additional functional annotations were performed using the biological databases COG (Cluster of Orthologous Groups) and KO (KEGG Orthology) by grouping genes into orthologous groups and functional categories, using the programs eggNOG, BlastKOALA (Huerta-Cepas et al., 2017), and antiSMASH (Blin et al., 2021). Phylogenetic analysis was performed on a complete-genome basis using the Type (Strain) Genome Server (https://tygs.dsmz.de) to determine species.

### 2.6 Maize inoculation with *Bacillus* strains under field conditions

To evaluate the impact of bacterial inoculants in maize, five field trials were conducted in different locations in Brazil: Location A - Piracicaba, SP (coordinates: 22°46’26.0”S 47°34’50.2”W); Location B - Boa Esperança do Sul, SP (coordinates: 21°55’59.0”S 48°17’47.0”W); Location C - Capela do Alto, SP (coordinates: 23°27’10.0”S 47°45’27.0”W); Locations D - Toledo, PR (Coordinates: 24°37’18.0”S 53°42’35.0”W); and Location E - Viçosa, MG (coordinates: 20°44’45.5”S 42°50’33.1”W). According to varietal recommendation by region, maize hybrids used were FS575 PWU (Forseed) in locations A, B and C, and BM990 VIP3 (Biomatrix) in locations D and E. All field experiments were sown in March 2024, harvested in July 2024, and maintained under non-irrigated conditions throughout the cycle. **Table 1** presents the physicochemical characteristics of the soils at the experimental fields. Soil analyses were performed prior to sowing by randomly collecting individual soil samples in the 0 to 20 cm of the topsoil. These samples were subsequently homogenized into a composite sample for each location to ensure representative characterization of local soil parameters.

**Table 1:** Chemical and granulometric characteristics of soil collected from the 0.0–0.2 cm layer from each experimental field (2024).

Characteristics	Parameters	Location A	Location B	Location C	Location D	Location E
Granulometric	Soil texture	Clay	Sandy loam	Clay loam	Clay	Clay
	Clay composition	535	260	360	466	460
	Silt composition	170	190	240	180	110
	Sand composition	295	550	400	354	430
Chemical	pH (CaCl_2_)	5.7	4.8	4.5	5.13	5.8
	V (%)	79	25.1	78.7	57.94	49.1
	P (mg/dm^3^)	35	18	28	26.22	21
	Organic matter (g/dm^3^)	28	9	11.2	24.18	33.3
	Ca^2+^ (cmol/dm^3^)	4.9	5	5.15	3.39	1.4
	Mg^2+^ (cmol/dm^3^)	2.7	2	4.26	1.94	0.9
	K^+^ (cmol/dm^3^)	0.3	0.5	0.2	0.63	0.35
	Al^3+^ (cmol/dm^3^)	0	0.2	0.1	0.08	0
	H+Al (cmol/dm^3^)	2.1	6.52	2.6	4.32	1.2
	Sum of Bases (cmol/dm^3^)	7.9	4.5	9.6	5.95	5.86
	Cation Exchange Capacity (cmol/dm^3^)	10	9.7	12.3	10.27	24.9

(P, K and micronutrients): Mehlich-1 extractor; (Al, Ca, Mg): KCl extractor 1 mol L^−1^; (H+Al): pH SMP (7.5); (pH): CaCl_2_ extractor 0.01 mol L^−1^.

The experiments were carried out using a randomized complete block design, with *k* = 5 treatments and *b* = 4 blocks, resulting in 20 experimental plots per location and 100 observations overall. Each location was analyzed separately. For a given location, let *X_ij_* denote the productivity (t/ha) associated with treatment *j* in block *i*, for *i* = 1*,…,b* and *j* = 1*,…,k*.

Each experimental plot consisted of 12 rows of maize, each 10 m long. The treatments comprised inoculation with each of three *Bacillus* strains (5D5, 6E9, and 1A11), formulated as commercial liquid inoculants by Simbiose company and supplied for the experiments. All inoculants were standardized to a viable cell concentration of 4.0 × 10^9^ CFU mL ^−1^ (Colony Forming Unit), and each strain was applied individually at a rate of 3 mL kg^−1^ of seed immediately before sowing. Additionally, an uninoculated control treatment, consisting of 0.85% (w/v) NaCl solution, was used as a negative control, while the commercial inoculant Auras^®^ formulated with a viable cell concentration of 1.0 × 10^8^ CFU mL^−1^ of *Bacillus aryabhattai* served as a positive control of performance, applied at a rate of 4 mL kg^−1^ of seed. After application, the seeds were allowed to air dry at room temperature for 5 minutes prior to sowing.

To establish a plant density of approximately 60,000 plants ha^−1^, 3.5 seeds were sown per linear meter of furrow, with 50 cm spacing between rows. The seed planting depth was set at 5 cm. During planting, granulated fertilizer (04–30–10 NPK plus 0.1 B + 0.2 Zn) was applied based on soil chemical analyses for each location, at rates of 200 to 350 kg ha^−1^, and placed 5 cm below the seeds. Additionally, between the V3 and V4 phenological stages, 100 kg ha^−1^ of urea (45% N) was applied as a nitrogen source. The experimental field followed management practices typical of commercial maize cultivation in Brazil, maintaining consistent cultural practices across treatments.

At the end of the growth cycle, maize plots were harvested, and grain yields were measured in tons per hectare (t ha^−1^) when grain moisture reached 13% (dry weight basis). Yield assessments were calculated from the average grain weight across experimental replicates for each treatment.

### 2.7 Weather data

Climatic information for all experimental fields was obtained from the NASA POWER database, following the approach described in (Monteiro et al., 2018), and covered the entire maize-growing season. The dataset was directly retrieved from Power (2024).

### 2.8 Statistical analysis

Statistical analyses were performed separately for each experiment. Data from the *in vitro* assays were subjected to analysis of variance (ANOVA) using SISVAR version 5.4 (Ferreira, 2019), and treatment means were compared using the Scott–Knott test at the 5% significance level.

For the plant experiments conducted under hydroponic conditions (with and without osmotic stress, evaluated as independent experiments), data were subjected to ANOVA, and adjusted means (estimated marginal means) were obtained. All pairwise comparisons between adjusted treatment means were performed using Tukey’s test at the 5% significance level (*p <* 0.05) with the emmeans package (Lenth, 2016) in software R (R Core Team, 2026).

Multivariate patterns were assessed using principal component analysis (PCA) after data standardization, with variance explained and variable contributions derived from eigenvalues using the factoextra package (Kassambara and Mundt, 2016), and visualized using biplots generated with ggplot2 (Wickham, 2016). Root system morphology traits were evaluated using radar plots based on min–max normalization (0– 1), with averages calculated according to inoculation and experimental conditions using dplyr (Wickham et al., 2026), and visualized with the fmsb package (Nakazawa, 2024).

Statistical analyses of the field experiment data were performed using the R software (R Core Team, 2024), with the agricolae package (De Mendiburu et al., 2015) employed for non-parametric tests and subsequent treatment grouping. Graphs were constructed using the ggplot2 package (Wickham, 2016). A significance level of 5% was adopted for all analyses.

The nonparametric Friedman test (Friedman, 1937; Conover, 1999) was employed, which is suitable for randomized block-design experiments when the assumptions of analysis of variance are not met. This test is based on the ranking of observations within each block.

Let *X_ij_*be the observation corresponding to treatment *j* in block *i*, for *i* = 1*,…,b* and *j* = 1*,…,k*. In each block, the observations are replaced by their respective ranks *R*(*X_ij_*), and the sums of the ranks for each treatment are calculated as:


$$R_{j}=\sum_{i=1}^{b}R(X_{ij}),\;j=1,\ldots ,k.$$

The Friedman test statistic is given by:


$$T_{1}=\frac{12}{bk(k+1)}\sum_{j=1}^{k}{\left(R_{j}-\frac{b(k+1)}{2}\right)}^{2},$$

which, under the null hypothesis of no treatment effect, has an approximate chi-square distribution with *k* −1 degrees of freedom (Conover, 1999).

When significant differences were detected (*p <* 0.05), multiple comparisons between treatments were performed based on rank sums, as described by Conover (1999).

## 3 Results

### 3.1 *Bacillus* strains show plant-growth-promoting mechanisms *in vitro*

A total of 414 bacterial strains were isolated from soil samples collected at different locations within the semi-arid Brazilian Caatinga biome in Ceará State. Among these, 28 isolates were capable of growing under reduced water activity conditions (*a_w_*= 0.897) when the culture medium was supplemented with sorbitol at 520 g L^−1^. Of these, 17 isolates tolerated higher sorbitol concentrations (780 g L^−1^), corresponding to a water activity of *a_w_*= 0.807 (**Table 2**). All 28 selected strains were deposited in the Embrapa Collection of Multifunctional and Phytopathogenic Microorganisms of Maize and Sorghum.

**Table 2:** Molecular identification and plant growth–promoting attributes of 28 selected *Bacillus* strains isolated from semi-arid soils of the Brazilian Caatinga biome.

Strain	CMMF number access	Molecular identification (16S rDNA)	Sorbitol growing capacity		EPS∗	BIO∗	SID∗	BNF∗	Phosphatase $\left(\mu {\text{g pNP mL}}^{-1}{\text{ min}}^{-1}\right)$		IAA $\left(\mu {\text{g mL}}_{-1}\right)$	${\text{Ca}}_{3}{\left({\text{PO}}_{4}\right)}_{2}$ solubilization $\left({\text{mg L}}^{-1}\right)$
			$520{\text{ g L}}^{-1}$	$780{\text{ g L}}^{-1}$					Acid	Alkaline		
1A11	CMPC 2388	*B. subtilis*	+∗∗	-∗∗∗	+	–	+	–	11.22^∗∗∗^	15.34*^f^*	14.19*^c^*	49.04
1C2	CMPC 2389	*B. subtilis*	+	+	+	+	+	+	10.89*^g^*	49.70*^d^*	30.59*^a^*	–
1H1	CMPC 2390	*B. subtilis*	+	+	+	+	+	+	17.51*^f^*	50.06*^d^*	29.63*^a^*	–
1H10	CMPC 2391	*B. subtilis*	+	+	+	+	+	+	8.43*^h^*	13.09*^f^*	30.54*^a^*	40.64
2A7	CMPC 2392	*B. subtilis*	+	–	+	+	+	–	14.80*^g^*	21.62*^f^*	32.87*^a^*	–
2A8	CMPC 2393	*B. subtilis*	+	–	+	+	+	–	13.26*^g^*	10.86*^f^*	24.34*^b^*	–
2B5	CMPC 2394	*B. subtilis*	+	–	+	+	+	–	14.02*^g^*	11.82*^f^*	25.54*^b^*	–
2B7	CMPC 2395	*B. subtilis*	+	+	+	+	+	+	36.51*^e^*	27.40*^e^*	23.09*^b^*	–
2B8	CMPC 2396	*B. subtilis*	+	–	+	+	+	+	6.41*^h^*	13.41*^f^*	17.99*^c^*	–
2C5	CMPC 2397	*B. subtilis*	+	+	+	+	+	+	38.30*^e^*	36.60*^e^*	30.00*^a^*	–
2C6	CMPC 2398	*B. subtilis*	+	–	+	+	+	+	40.09*^e^*	29.58*^e^*	28.04*^a^*	–
2D7	CMPC 2399	*B. subtilis*	+	–	+	+	+	+	54.11*^c^*	40.09*^e^*	31.37*^a^*	–
2E5	CMPC 2400	*B. subtilis*	+	–	+	+	+	+	18.53*^f^*	23.97*^f^*	28.01*^a^*	–
2E7	CMPC 2401	*B. subtilis*	+	–	+	+	+	+	18.83*^f^*	13.44*^f^*	14.34*^c^*	41.39
2F6	CMPC 2402	*B. subtilis*	+	+	+	+	+	+	46.22*^d^*	67.11*^c^*	26.81*^b^*	–
2F7	CMPC 2403	*B. subtilis*	+	+	+	+	+	+	46.19*^d^*	65.27*^c^*	23.33*^b^*	–
2G5	CMPC 2404	*B. subtilis*	+	–	+	+	+	+	89.53*^b^*	81.56*^b^*	26.69*^b^*	–
2G7	CMPC 2405	*B. subtilis*	+	–	+	+	+	+	56.03*^c^*	41.44*^e^*	26.69*^b^*	–
3C4	CMPC 2406	*B. amyloliquefaciens*	+	–	+	+	+	–	16.54*^f^*	17.87*^f^*	24.95*^b^*	–
3D4	CMPC 2407	*B. amyloliquefaciens*	+	+	+	+	+	+	6.44*^h^*	13.94*^f^*	22.60*^b^*	–
3G4	CMPC 2408	*B. subtilis*	+	+	+	+	+	+	8.78*^h^*	18.39*^f^*	20.98*^b^*	–
3H4	CMPC 2409	*B. amyloliquefaciens*	+	+	+	+	+	+	5.51*^h^*	13.94*^f^*	24.17*^b^*	–
5A6	CMPC 2410	*B. subtilis*	+	+	+	+	+	–	4.51*^h^*	11.89*^f^*	38.75*^a^*	–
5D5	CMPC 2411	*B. velezensis*	+	+	+	+	+	+	16.05*^f^*	19.09*^f^*	29.12*^a^*	42.02
6C12	CMPC 2412	*B. amyloliquefaciens*	+	+	+	+	+	–	13.41*^g^*	17.77*^f^*	31.40*^a^*	–
6D3	CMPC 2413	*B. amyloliquefaciens*	+	+	+	+	+	–	13.50*^g^*	20.06*^f^*	24.22*^b^*	–
6D11	CMPC 2414	*B. amyloliquefaciens*	+	+	+	+	+	+	13.73*^g^*	20.00*^f^*	22.25*^b^*	–
6E9	CMPC 2415	*B. velezensis*	+	+	+	+	+	+	12.80*^g^*	21.29*^f^*	16.42*^c^*	69.84

^∗^ EPS, Exopolysaccharide; BIO, Biofilm; SID, Siderophore (carboxylate); BNF, Biological nitrogen fixation; IAA, Indole-3 acetic acid. ^∗∗^+ indicates positive activity; ^∗∗∗^- indicates absence of activity. ^∗∗∗∗^Means followed by the same letter do not differ significantly at the 5% level by the Scott-Knott test. CMMF, Embrapa Collection of Multifunctional and Phytopathogenic Microorganisms of Maize and Sorghum.

Molecular identification based on partial *16S rRNA* gene sequencing revealed that isolates belonged to *Bacillus subtilis*, *Bacillus amyloliquefaciens*, and *Bacillus velezensis* (**Table 2**).

Although all selected strains expressed multiple plant growth–promoting traits, marked quantitative differences were observed among isolates (**Table 2**). Exopolysaccharide (EPS) production and siderophore synthesis were uniformly detected, whereas 75% exhibited biofilm-forming capacity and 64% were capable of atmospheric nitrogen fixation. Indole-3-acetic acid (IAA) synthesis, using tryptophan as a metabolic precursor, was detected in all isolates, with concentrations ranging from 14.19 *µ*g mL^−1^ to 38.75 *µ*g mL^−1^ (**Table 2**).

Phosphatase activity further differentiated the isolates. Strain 2G5 showed the highest acid and alkaline phosphatase activities (89.53 and 81.56 *µ*g pNP mL^−1^ min^−1^), while strains 2D7, 2F6, 2F7, and 2G7 displayed moderate enzymatic activity. In addition, the solubilization capacity of tricalcium phosphate [Ca_3_(PO_4_)_2_] in a subset of five selected strains ranged from 40.64 to 69.84 mg L^−1^ (**Table 2**).

### 3.2 Inoculation with *Bacillus* strains promotes plant growth and induces tolerance to osmotic stress in maize seedlings

Based on the evaluation of plant-growth-promoting traits *in vitro*, 12 promising *Bacillus* strains were selected for evaluation under osmotic stress induced by polyethylene glycol (PEG 6000) in a hydroponic system. Inoculated maize seedlings exhibited pronounced, strain-dependent alterations in root system morphology, chlorophyll content, and biomass accumulation compared with the uninoculated control (**Tables 3**, **4**).

**Table 3:** Adjusted means for root morphology traits, chlorophyll content and dry weight of maize seedlings inoculated with *Bacillus* strains grown under hydroponic conditions with osmotic stress induced by polyethylene glycol 6000.

Strain	L	SA	SA1	SA2	SA3	D	V	SDW	RDW	TDW	SPAD
Non-inoculated^∗^	292.18*^d^*	92.13*^a^*	34.30*^c^*	32.85*^ef^*	22.51*^cd^*	1.04*^abc^*	2.33*^cd^*	0.23*^bc^*	0.29*^d^*	0.52*^cd^*	24.01*^c^*
B119^∗∗^	420.53*^a^*	120.65*^a^*	51.82*^ab^*	40.59*^abc^*	26.22*^abc^*	0.91*^ef^*	2.77*^ab^*	0.31*^ab^*	0.31*^cd^*	0.62*^b^*	24.26*^c^*
B2084^∗∗^	284.61*^bcde^*	85.10*^bcde^*	31.84*^defg^*	33.16*^cdef^*	20.01*^cd^*	0.99*^abcd^*	2.06*^de^*	0.11*^d^*	0.36*^abcd^*	0.47*^d^*	24.39*^abc^*
6E9	307.87*^bcd^*	91.14*^bcd^*	37.68*^bcdef^*	30.12*^ef^*	22.72*^bcd^*	0.98*^abcde^*	2.18*^de^*	0.14*^cd^*	0.36*^abcd^*	0.50*^cd^*	25.88*^abc^*
5D5	218.77*^de^*	74.32*^cde^*	21.61*^fg^*	31.80*^ef^*	22.47*^bcd^*	1.09*^ab^*	2.06*^de^*	0.14*^cd^*	0.40*^abc^*	0.54*^bcd^*	26.05*^abc^*
1H10	209.86*^de^*	70.12*^de^*	19.61*^g^*	30.42*^ef^*	21.97*^bcd^*	1.09*^a^*	1.92*^de^*	0.09*^d^*	0.42*^ab^*	0.50*^cd^*	23.88*^c^*
2E7	180.89*^e^*	61.66*^e^*	20.40*^g^*	25.16*^f^*	19.76*^d^*	1.07*^ab^*	1.70*^e^*	0.12*^d^*	0.36*^abcd^*	0.48*^cd^*	25.13*^abc^*
1A11	420.87*^a^*	121.49*^a^*	48.46*^abcde^*	46.71*^a^*	22.02*^bcd^*	0.95*^cde^*	2.81*^abc^*	0.34*^a^*	0.38*^abcd^*	0.72*^a^*	25.95*^abc^*
6D3	373.19*^abc^*	106.97*^abc^*	45.47*^abcde^*	37.21*^abcde^*	23.05*^bcd^*	0.90*^cde^*	2.46*^bcd^*	0.27*^abc^*	0.30*^bcd^*	0.57*^bcd^*	24.82*^bc^*
6C12	422.43*^ab^*	116.32*^ab^*	53.81*^ab^*	37.54*^abcde^*	22.81*^cd^*	0.85*^e^*	2.56*^abcd^*	0.30*^abc^*	0.26*^d^*	0.56*^bcd^*	21.98*^c^*
2B5	386.13*^abc^*	109.09*^ab^*	48.06*^abcde^*	36.22*^bcde^*	22.09*^cd^*	0.90*^de^*	2.47*^bcd^*	0.27*^abc^*	0.28*^cd^*	0.54*^bcd^*	24.65*^c^*
2G5	459.83*^a^*	132.60*^a^*	60.86*^a^*	42.13*^abc^*	29.71*^ab^*	0.90*^de^*	3.06*^a^*	0.31*^ab^*	0.28*^cd^*	0.59*^bc^*	28.88*^ab^*
2B8	411.67*^ab^*	115.67*^ab^*	50.95*^abcd^*	38.55*^abcd^*	24.84*^bcd^*	0.90*^de^*	2.60*^abcd^*	0.28*^abc^*	0.28*^cd^*	0.56*^bcd^*	25.18*^abc^*
2C5	472.40*^a^*	132.19*^a^*	59.12*^ab^*	44.48*^ab^*	32.20*^a^*	0.87*^de^*	2.96*^ab^*	0.32*^ab^*	0.25*^d^*	0.58*^bc^*	29.02*^a^*
2A8	400.47*^ab^*	112.64*^ab^*	49.54*^abcde^*	38.20*^abcde^*	23.89*^bcd^*	0.89*^de^*	2.54*^abcd^*	0.27*^abc^*	0.28*^cd^*	0.55*^bcd^*	23.68*^c^*

^∗^ Uninoculated control.

^∗∗^ Positive control.

^∗∗∗^ Adjusted means followed by the same lower case letters indicate not significant difference between strains by pairwise Tukey’s test (*p <* 0.05) L, total root length (cm); SA, total surface area (cm^2^); D, average diameter (mm); V, total root volume (cm^3^); SA1, surface area of roots with diameter between 0 and 1 mm (cm^2^); SA2, surface area of roots with diameter between 1 and 2 mm (cm^2^); SA3, surface area of roots with diameter *>* 2 mm (cm^2^); SDW, shoot dry.

weight (g), RDW, root dry weight (g); TDW, total dry weight (g); SPAD, relative chlorophyll content.

**Table 4:** Adjusted means for root morphology traits, chlorophyll content and dry weight of inoculated maize seedlings grown under hydroponic conditions without osmotic stress.

Strain	L	SA	SA1	SA2	SA3	D	V	SDW	RDW	TDW	SPAD
Non-inoculated^∗^	544.74*^ab^*	147.83*^ab^*	62.07*^abcd^*	48.34*^bc^*	38.67*^a^*	0.86*^c^*	3.23*^abc^*	0.34*^ab^*	0.26*^bc^*	0.59*^a^*	27.18*^bc^*
B119^∗∗^	585.91*^ab^*	153.61*^ab^*	73.59*^ab^*	46.86*^bc^*	31.89*^abcdef^*	0.84*^abc^*	3.24*^abc^*	0.35*^ab^*	0.22*^d^*	0.56*^ab^*	28.24*^ab^*
B2084^∗∗^	559.22*^ab^*	140.23*^ab^*	68.15*^abc^*	47.25*^bc^*	24.33*^def^*	0.81*^c^*	2.80*^bcd^*	0.24*^ab^*	0.37*^a^*	0.62*^a^*	27.45*^abc^*
6E9	461.06*^bc^*	120.01*^bcd^*	61.01*^abcd^*	39.21*^bcd^*	22.54*^def^*	0.87*^abc^*	2.52*^cde^*	0.29*^b^*	0.28*^bc^*	0.57*^ab^*	27.76*^abc^*
5D5	504.22*^abc^*	128.49*^bcd^*	67.14*^abc^*	41.02*^bcd^*	21.67*^ef^*	0.83*^abc^*	2.61*^bcde^*	0.27*^b^*	0.31*^b^*	0.58*^ab^*	28.08*^abc^*
1H10	343.97*^c^*	91.08*^d^*	41.65*^d^*	35.05*^d^*	20.32*^f^*	0.92*^a^*	1.98*^e^*	0.12*^c^*	0.42*^a^*	0.54*^ab^*	27.75*^abc^*
2E7	343.58*^c^*	96.48*^d^*	46.80*^cd^*	34.98*^d^*	21.33*^ef^*	0.92*^a^*	2.21*^de^*	0.11*^d^*	0.37*^a^*	0.48*^b^*	27.65*^abc^*
1A11	654.85*^a^*	172.00*^a^*	83.77*^a^*	60.47*^a^*	23.82*^cdef^*	0.83*^bc^*	3.62*^a^*	0.43*^a^*	0.21*^d^*	0.64*^a^*	27.62*^abc^*
6D3	507.58*^abc^*	138.05*^abcd^*	61.35*^abcd^*	42.36*^bcd^*	30.25*^abcdef^*	0.86*^abc^*	3.02*^abcd^*	0.34*^ab^*	0.23*^cd^*	0.58*^ab^*	27.78*^abc^*
6C12	555.97*^ab^*	155.13*^ab^*	68.23*^abcd^*	49.85*^ab^*	38.67*^a^*	0.89*^abc^*	3.47*^abc^*	0.37*^ab^*	0.20*^d^*	0.57*^ab^*	29.38*^a^*
2B5	514.89*^abc^*	142.91*^abc^*	61.30*^abcd^*	45.49*^bcd^*	33.52*^abcdef^*	0.87*^abc^*	3.17*^abcd^*	0.32*^ab^*	0.23*^cd^*	0.56*^ab^*	28.01*^ab^*
2G5	579.87*^ab^*	159.99*^ab^*	73.32*^ab^*	46.30*^bcd^*	36.78*^abc^*	0.90*^ab^*	3.53*^ab^*	0.35*^ab^*	0.21*^d^*	0.56*^ab^*	28.48*^ab^*
2B8	587.42*^ab^*	160.00*^ab^*	73.39*^ab^*	48.42*^abcd^*	36.55*^abcd^*	0.88*^abc^*	3.49*^abc^*	0.33*^ab^*	0.22*^cd^*	0.55*^ab^*	27.94*^ab^*
2C5	509.08*^abc^*	141.72*^abc^*	63.78*^abcd^*	42.30*^bcd^*	31.63*^abcdef^*	0.88*^abc^*	3.15*^abcd^*	0.35*^ab^*	0.21*^cd^*	0.56*^ab^*	29.38*^a^*
2A8	434.76*^bc^*	125.45*^abcd^*	52.82*^bcd^*	37.95*^cd^*	27.21*^bcdef^*	0.91*^ab^*	2.88*^abcde^*	0.31*^ab^*	0.23*^cd^*	0.54*^ab^*	25.71*^c^*

^∗^ Uninoculated control.

^∗∗^ Positive control.

^∗∗∗^ Adjusted means followed by the same lower case letters indicate not significant difference between strains by pairwise Tukey’s test (*p <* 0.05) L, total root length (cm); SA, total surface area (cm^2^); D, average diameter (mm); V, total root volume (cm^3^); SA1, surface area of roots with diameter between 0 and 1 mm (cm^2^); SA2, surface area of roots with diameter between 1 and 2 mm (cm^2^); SA3, surface area of roots with diameter *>* 2 mm (cm^2^); SDW, shoot dry weight (g); RDW, root dry weight (g); TDW, total dry weight (g); SPAD, relative chlorophyll content.

**Figure 1:**
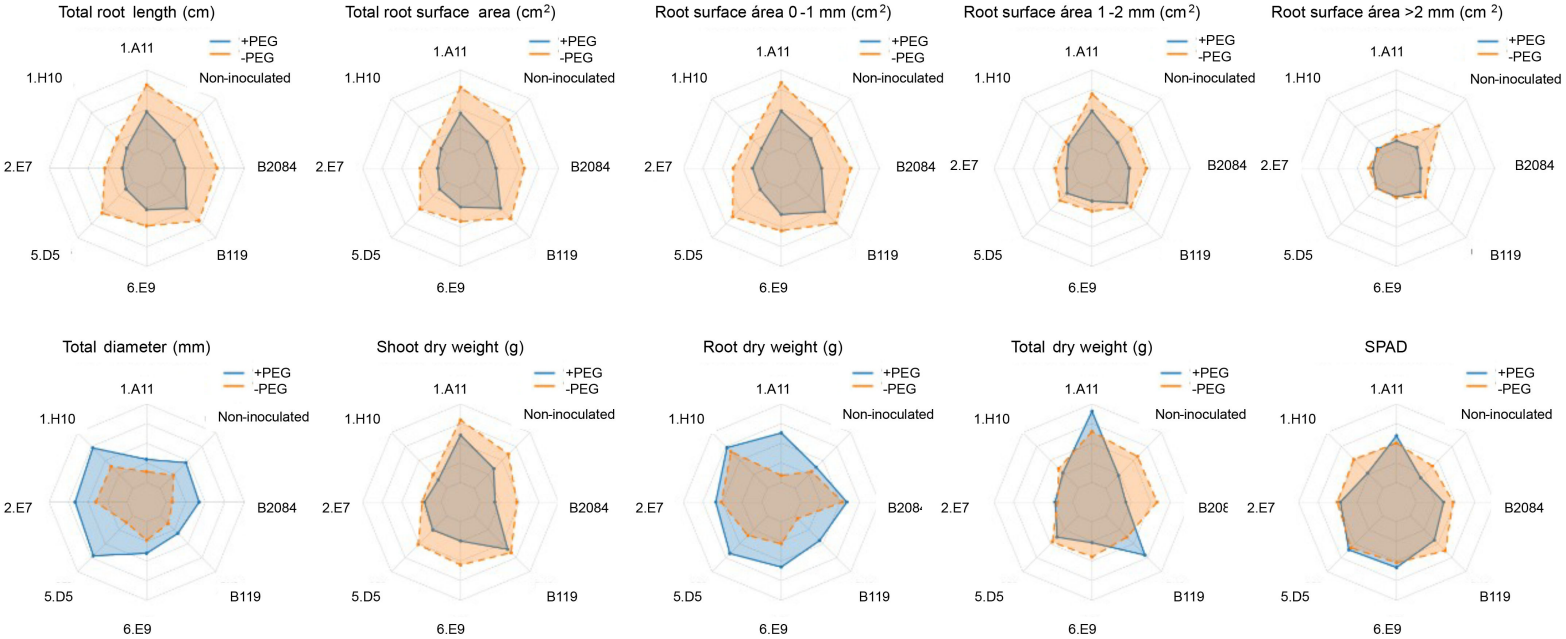
Phenotypic means among clusters within *Bacillus* strain groups (B119, B2084, 1A11, 6E9, 5D5, 2E7, and 1H10) grown under hydroponic conditions with and without osmotic stress induced by polyethylene glycol (PEG 6000). The evaluated traits included total root length, total root surface area, root surface area (0–1 mm), root surface area (1–2 mm), root surface area (*>* 2 mm), average root diameter, shoot dry weight, root dry weight, total dry weight, and relative chlorophyll content (SPAD). Phenotypic data were centered as described in **Table 3**.

Under PEG-induced stress, inoculated seedlings exhibited increases in total root length, total root surface area, fine root surface area, and root volume. Strain 1A11 induced the most consistent and balanced response, promoting significant increases in total root length, fine root surface area, root volume, and shoot, root, and total dry weight relative to the uninoculated control. Strain 6C12 significantly increases root length and surface area of fine roots. In contrast, strains 2C5 and 2G5 induced a distinct phenotype characterized by enhanced relative chlorophyll content (highest SPAD index) and increased root length, volume, and surface area among treatments (**Figure 1**; **Table 3**).

In the absence of osmotic stress, inoculation effects remained detectable but differed among strains. Strain 1A11 continued to outperform other treatments, significantly increasing total root length, total and fine root surface area, shoot dry weight, and total biomass, demonstrating growth-promoting capacity independent of stress conditions. Additionally, strains 6C12 and 2C5 increased chlorophyll content under non-stressed conditions, whereas strains 1H10 and 2E7 exerted more modest effects, primarily influencing root biomass and root diameter (**Figure 1**; **Table 4**).

**Figure 2:**
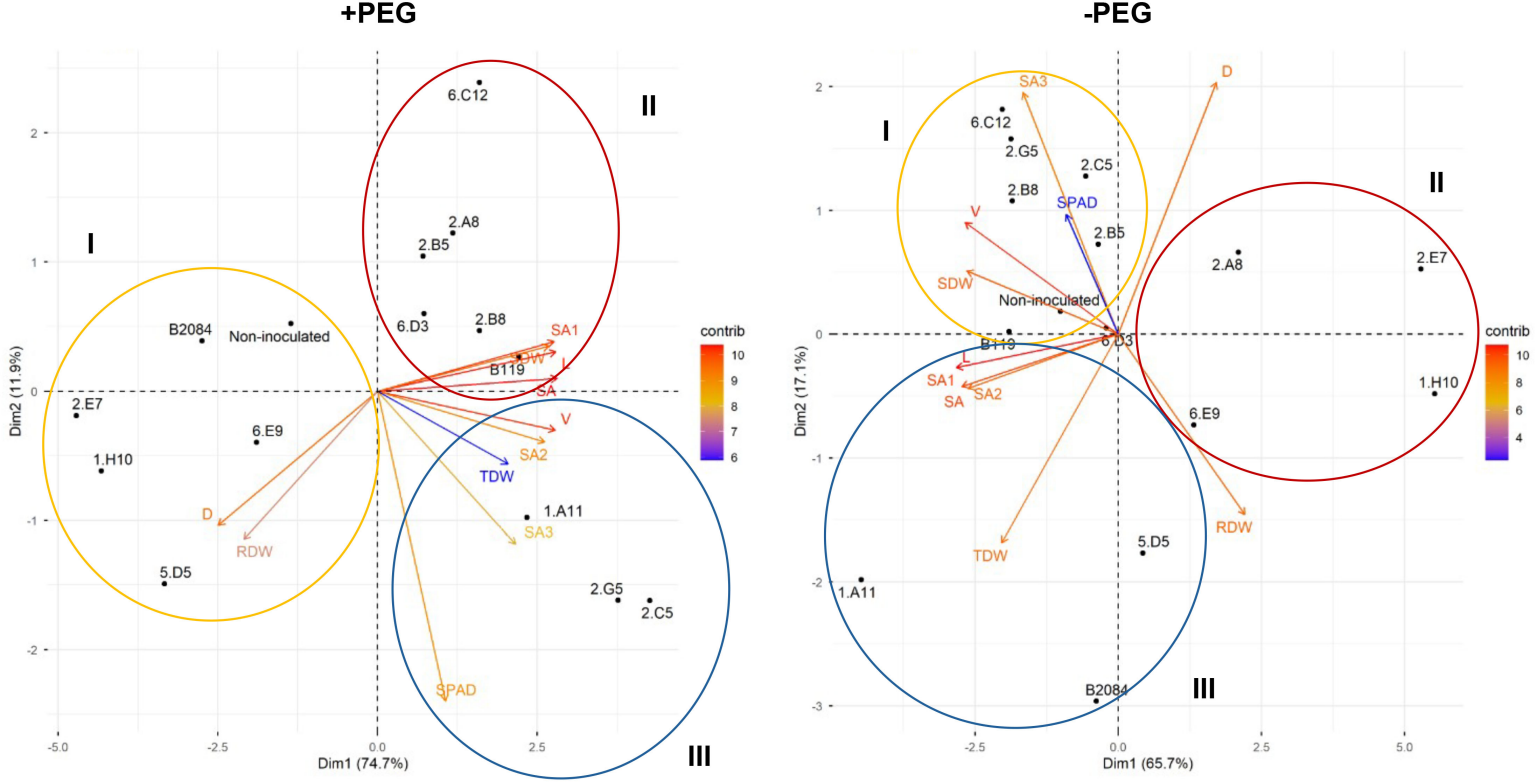
Principal component analysis (PCA) for root morphology and biomass-related traits. L, total root length; SA, total surface area; D, average diameter; V, total root volume; SA1, surface area of roots with diameter between 0 and 1 mm; SA2, surface area of roots with diameter between 1 and 2 mm; SA3, diameter of surface area of roots (*>* 2 mm); SDW, shoot dry weight; RDW, root dry weight; TDW, total dry weight; SPAD, relative chlorophyll content. The proportion of the total variance explained by each principal component (PC1 and PC2) is shown on their respective axis. Prior to PCA, traits were standardized to have the same normal distribution, with mean and variance equal to zero and one, respectively. Strains identifications are presented on **Table 2**.

Principal component analysis (PCA) was used to integrate root morphological traits, biomass accumulation, and physiological parameters under stressed and non-stressed conditions (**Figure 2**).

Under osmotic stress, the first two principal components explained 74.7% (PC1) and 11.9% (PC2) of the total phenotypic variability (**Figure 2**). Strain 1A11, 2G5, and 2C5 occupied a distinct position and were simultaneously associated with fine root surface area, root volume, SPAD index, and total biomass accumulation, indicating a multifunctional growth-promoting profile, whereas strains 1H10, 5D5, 2E7, and 6E9 clustered with root diameter and root dry weight.

Under non-stressed conditions, PC1 and PC2 explained 65.7% and 17.1% of the phenotypic variation, respectively (**Figure 2**). Strain 1A11 showed the strongest association with total dry weight and root surface area, while strains 1H10, 2E7, 5D5, 2A8, and 6E9 were more strongly associated with root dry weight.

**Figure 3:**
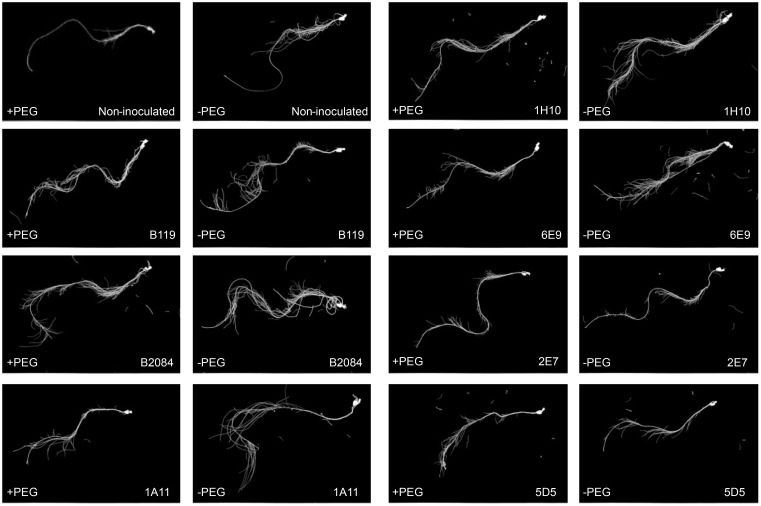
Representative images of maize roots non-inoculated and inoculated with different *Bacillus* strains (B119, B2084, 1A11, 1H10, 6E9, 2E7, and 5D5) grown under hydroponic conditions without osmotic stress (–PEG) and with osmotic stress induced by polyethylene glycol (+PEG). Images illustrate differences in root system architecture among treatments. Strain identifications are presented in **Table 2**.

**Figure 3** shows the root structure of maize inoculated with these five strains grown under hydroponic conditions compared to the uninoculated control and two experimental control strains, B119 and B2084 (**Figure 3**).

Since strains 1A11, 1H10, 2E7, 5D5, and 6E9 demonstrated superior performance under hydroponic conditions, both with and without osmotic stress, they were selected for genome sequencing.

### 3.3 Genomic characterization of genes related to PGP and drought stress conditions

**Figure 4:**
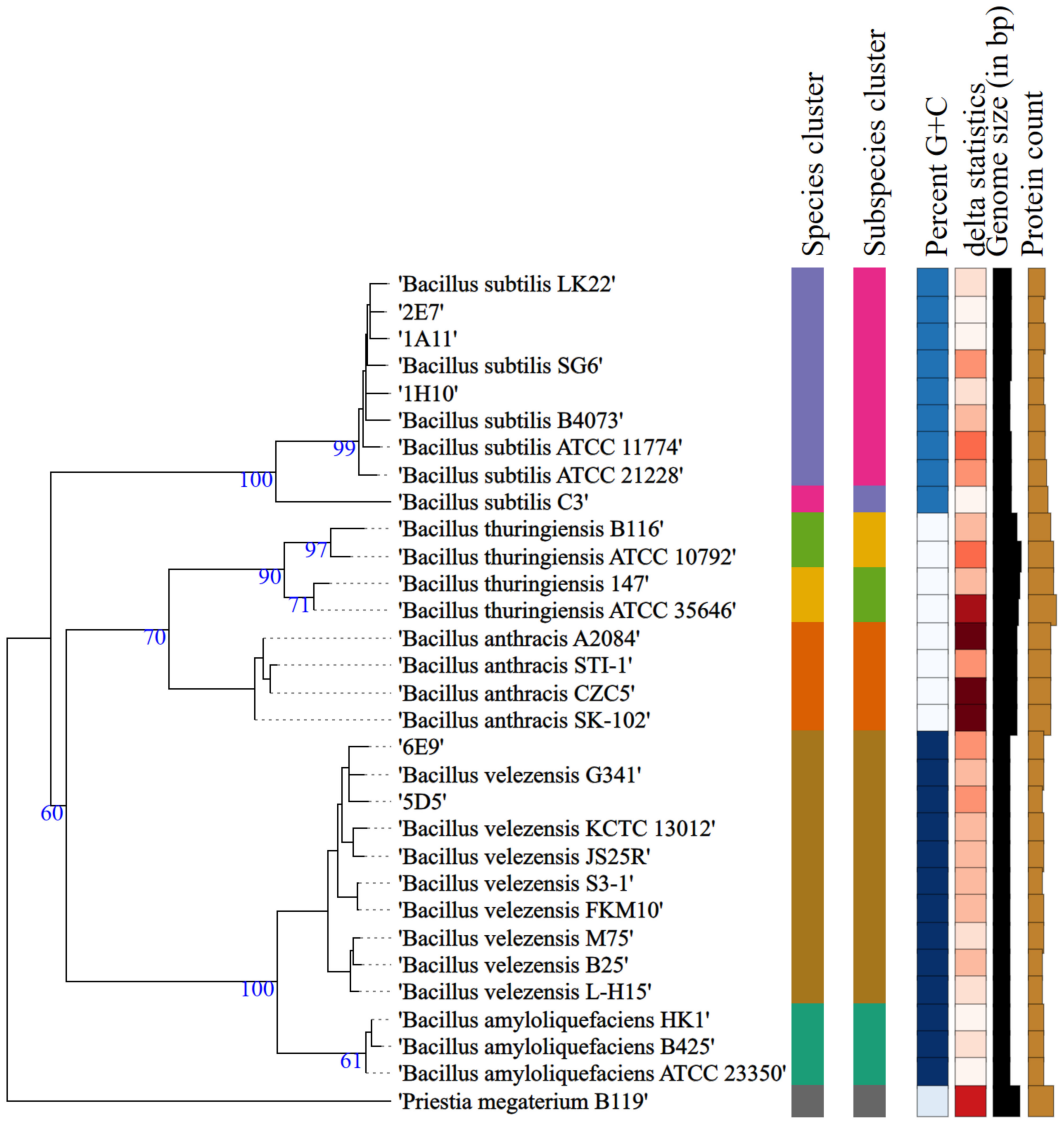
Phylogenetic tree generated with the TYGS software, based on the complete genome, showing the relationship between species of the genus *Bacillus*. Strains 6E9 and 5D5 grouped with the species B. velezensis, while strains 2E7, 1A11 and 1H10 grouped with the species *B. subtilis*. The columns, from left to right, represent the groups of the same species and subspecies (color variation for each group), percentage of GC content (variation of color intensity, becoming more intense as the percentage increases), delta statistics for evaluating phylogenetic accuracy in terms of similarity (variation of color intensity, becoming more intense as the index increases; the lower the delta value, the higher the accuracy), genome size (variation of the size of the horizontal bar as the genome size increases), and protein count (variation of the size of the horizontal bar as the protein count increases).

The molecular phylogenetic tree based on the complete genome clustered strains 6E9 and 5D5 with *Bacillus velezensis*, while strains 2E7, 1A11, and 1H10 clustered with the *B. subtilis* species (**Figure 4**).

The draft genomes and key genomic features of these five strains were summarized by Silva Ferreira et al. (2025). Whole-genome sequencing and functional annotation of these genomes revealed more than 200 conserved genes associated with PGP and drought stress resilience, including osmotic stress response, oxidative stress tolerance, phytohormone production, phosphorus solubilization, organic acid production, nitrogen metabolism, iron metabolism and acquisition, exopolysaccharide production, synthesis of organic and inorganic volatile compounds, motility, and chemotaxis (see **Supplementary Table 1**).

**Figure 5:**
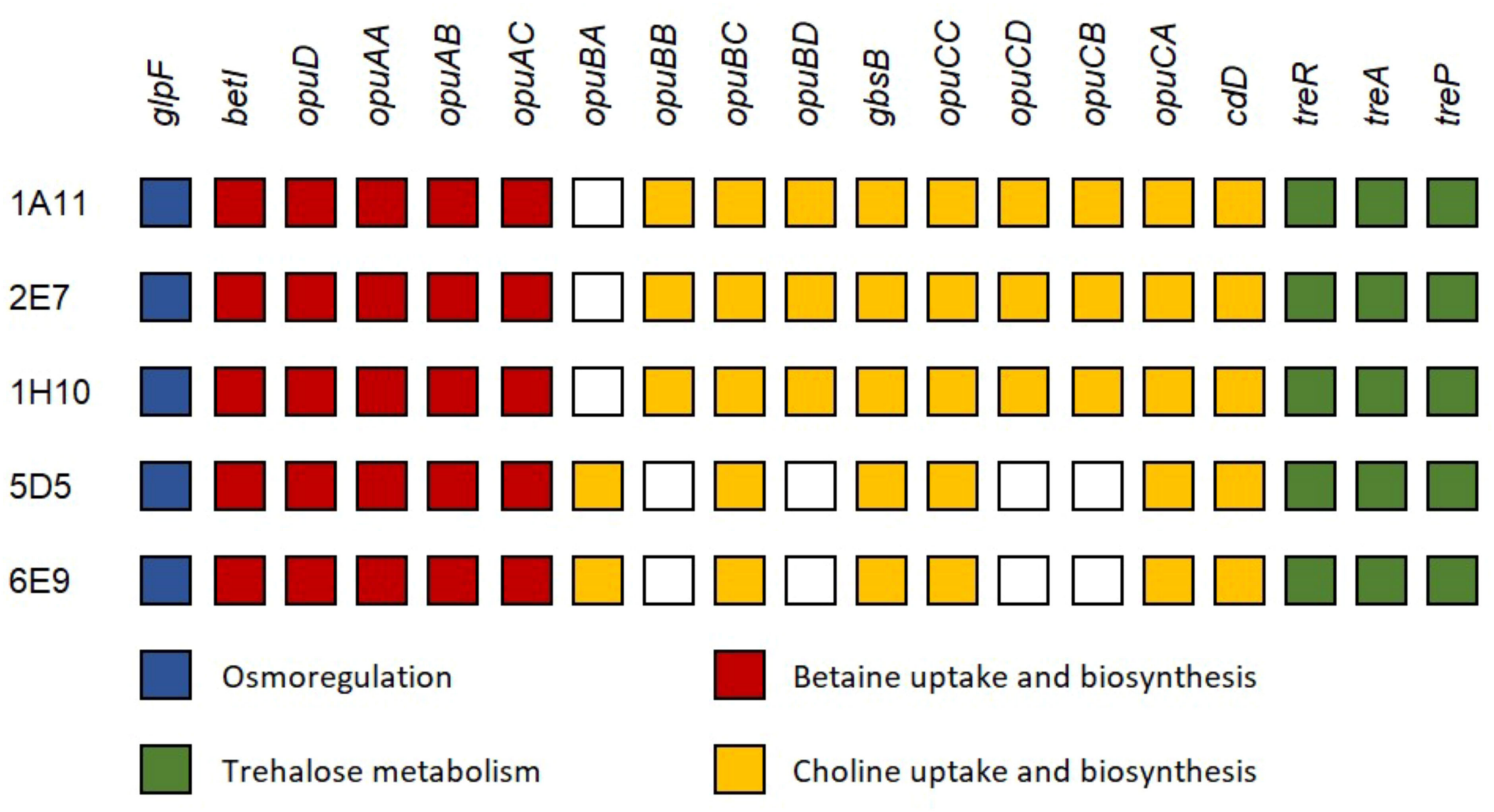
Presence-absence profile of genes identified in the genomes of strains 6E9, 5D5, 1H10, 2E7, and 1A11 associated with choline and betaine uptake, osmoregulation, and trehalose metabolism. Gene presence is indicated by colored squares, whereas absence is indicated by white squares.

Regarding genomic GC content, higher GC percentages were observed in strains identified as *B. velezensis*, which also had fewer protein-coding sequences and lacked the complete operon for choline biosynthesis and uptake due to the absence of the *opuBB* gene. Other plant growth-promoting genes absent in *B. velezensis* but present in *B. subtilis* strains included *ywh* and *skf* (secondary metabolite production), *ahpC* (oxidative stress), *efeU* and *ddc* (iron metabolism and acquisition), *glgC* and *glgP* (EPS production), *kduD* (organic acid metabolism), *aofH* (phytohormone production), and *nplT* (drought stress response) highlighting intra-species genomic variability related to stress adaptation (**Figure 5**; **Supplementary Table 1**).

Genes related to osmotic stress (opu operons, e.g., *opuA*, *opuB*, *opuC*), oxidative stress genes (*sod1*, *sod2*, *cat*, *dps*, *rex*, *fur*, *zurR*, *ahpC*, *ahpF*), and biosynthesis and transport of compatible solutes, such as *betI*, *gbsB*, and *cdD* (involved in glycine betaine and choline metabolism), along with the *rsb* operon that controls the activation of the alternative sigma factor B (*σ^B^*), a global regulator that promotes the expression of over 300 genes associated with stress responses in *Bacillus*, were detected in all genomes (Zhao et al., 2024). Genes involved in EPS production (*glg* and *eps* operons) and iron acquisition (bacillibactin synthesis and heme uptake) were also detected (see **Supplementary Table 1**).

All genomes contained tryptophan biosynthesis genes, including aldehyde dehydrogenase, monoamine oxidase, and the *trpAF* cluster, suggesting potential for indole-3-acetic acid (IAA) synthesis. Phosphorus metabolism genes were also conserved, including the *pst* and *pho* operons (high-affinity phosphate uptake), *pitA* (low-affinity transport), *ppaC*, *ppaX*, and *yjbB* (intracellular phosphate homeostasis), alongside organic acid biosynthesis genes (*gnt*, *lld*, *prp* operons). All genomes contained extensive secondary metabolite biosynthetic gene clusters, including those encoding surfactin, fengycin, and bacillibactin, thereby reinforcing their capacity to interact with microbes and modulate plant responses (see **Supplementary Table 1**).

### 3.4 Field performance identifies elite drought-mitigating strains

Based on their multifunctional plant growth-promoting (PGP) traits, hydroponic assays, and genomic analyses, three *Bacillus* strains (1A11, 5D5, and 6E9) were selected for field evaluation. An uninoculated control and a treatment with a commercial standard microbiological inoculant available on the market and recognized as a benchmark for mitigating water deficit (Castelo Sousa et al., 2023) were also included. The field trials were conducted in 2024 during the second maize crop season at five locations in Brazil, as described in the Material and Methods section.

**Figure 6:**
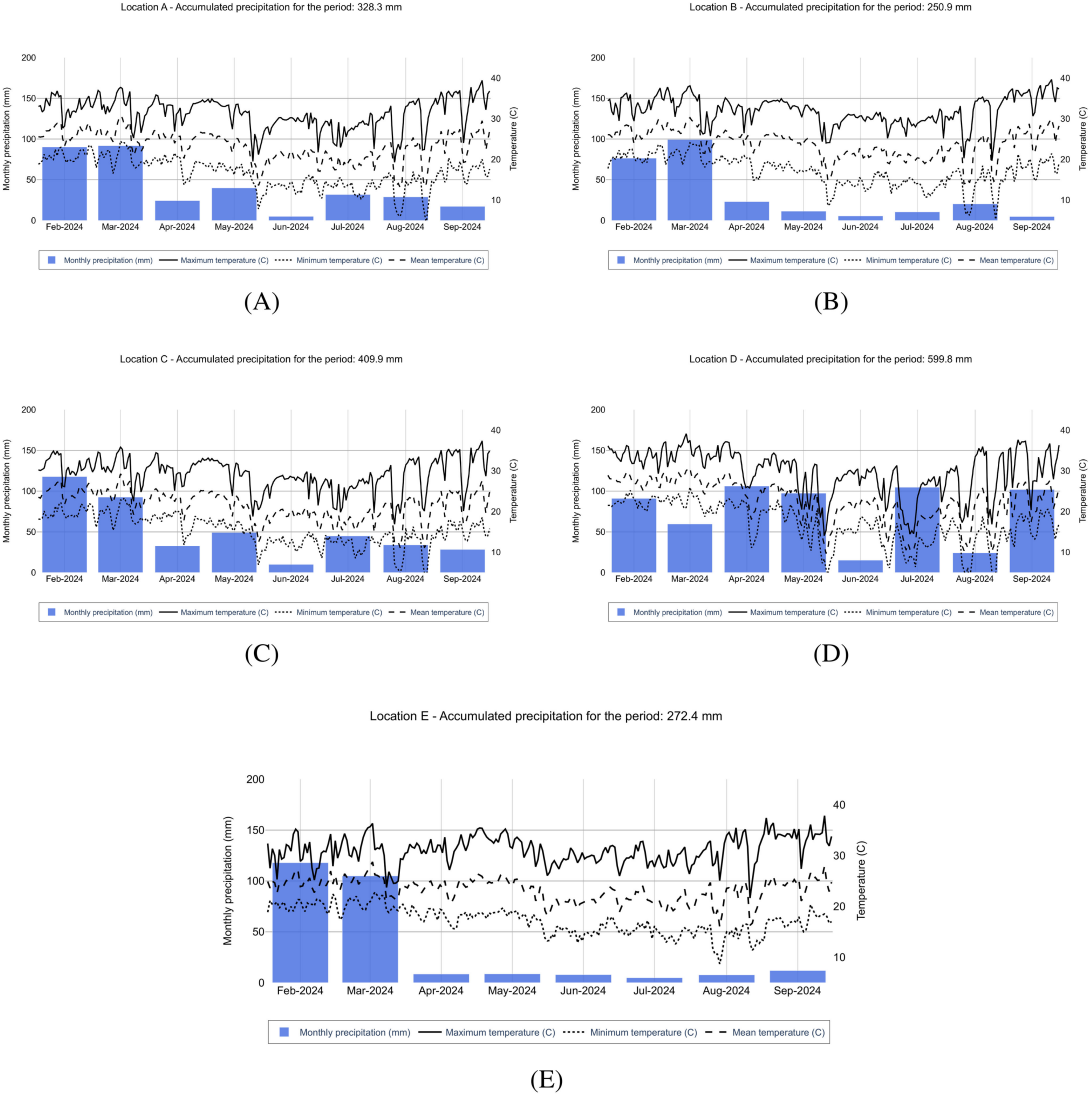
**(A–E)** Monthly precipitation and average temperature patterns during the experimental period for the five experimental sites. The bars represent the accumulated monthly precipitation (mm), while the lines indicate the maximum (Tmax), average (Tmean), and minimum (Tmin) temperatures (°C). Source: Nasa Power (Power, 2024).

**Figure 6** illustrates the temperature and precipitation patterns across the five experimental locations where field trials were conducted to assess the effects of bioinputs in maize performance. The climatic variability observed across locations played a central role in shaping maize productivity and in modulating the magnitude of the inoculation response. In particular, Location B represented a scenario of severe water limitation, with irregular rainfall distribution, the lowest accumulated rainfall (250.9 mm), and pronounced thermal stress. Under these conditions, yield potential was strongly constrained, highlighting the importance of stress-mitigating strategies. In contrast, although Location D also exhibited relatively low total precipitation (272.4 mm), the more favorable distribution of rainfall, especially during early crop establishment, likely contributed to improved stand development and partial buffering of the effects of later drought. Locations with cumulative precipitation above 300 mm presented more favorable moisture availability during critical phenological stages, resulting in overall higher productivity.

The results of physicochemical analysis of the soil indicated in **Table 1** demonstrated substantial variability in soil texture among the experimental fields, ranging from sandy loam in Location B and clay loam in Location C to predominantly clayey soils in Locations A, D, and E. Additionally, significant differences in chemical properties were observed, indicating variability in soil fertility status, particularly in pH, organic matter content, and macronutrient availability.

Despite this environmental heterogeneity, all strains, including the *B. aryabhattai*, which is the bacterial species present in the commercial inoculant formulation, consistently outperformed the uninoculated control across sites, demonstrating the robustness of microbial inoculation as a strategy to enhance maize performance under water-limited conditions. Notably, this consistent superiority across contrasting environments indicates that the selected strains were able to translate their functional traits into agronomic benefits under field conditions.

The observed gains can be interpreted as the result of multiple, complementary mechanisms. Under moderate stress conditions, improved root system morphology and nutrient acquisition likely played a dominant role in enhancing water uptake and sustaining plant growth. Under more severe drought, additional mechanisms such as rhizosphere hydration mediated by exopolysaccharides, osmotic adjustment, and oxidative stress mitigation may have become increasingly relevant. This context-dependent expression of benefits helps explain the stable performance of the strains across environments with distinct stress intensities. Importantly, the comparable or superior performance of the selected strains relative to the commercial inoculant, formulated with *B. aryabhattai*, reinforces their potential as competitive bioinoculants. While the commercial product is already recognized for drought mitigation, the Caatingaderived strains are consistently at least as effective, suggesting that their ecological origin under chronic water limitation may confer adaptive advantages in rain-fed agricultural systems. Overall, these results provide strong evidence that integrating ecologically adapted microbial resources with multi-environment field validation is an effective strategy for identifying elite bioinoculants. The ability of strains 1A11, 5D5, and 6E9 to maintain consistent performance across diverse climatic conditions highlights their potential to enhance yield stability and resilience in maize production systems facing increasing rainfall variability.

Statistical analysis of maize inoculation with *Bacillus* strains under field conditions.

**Figure 7:**
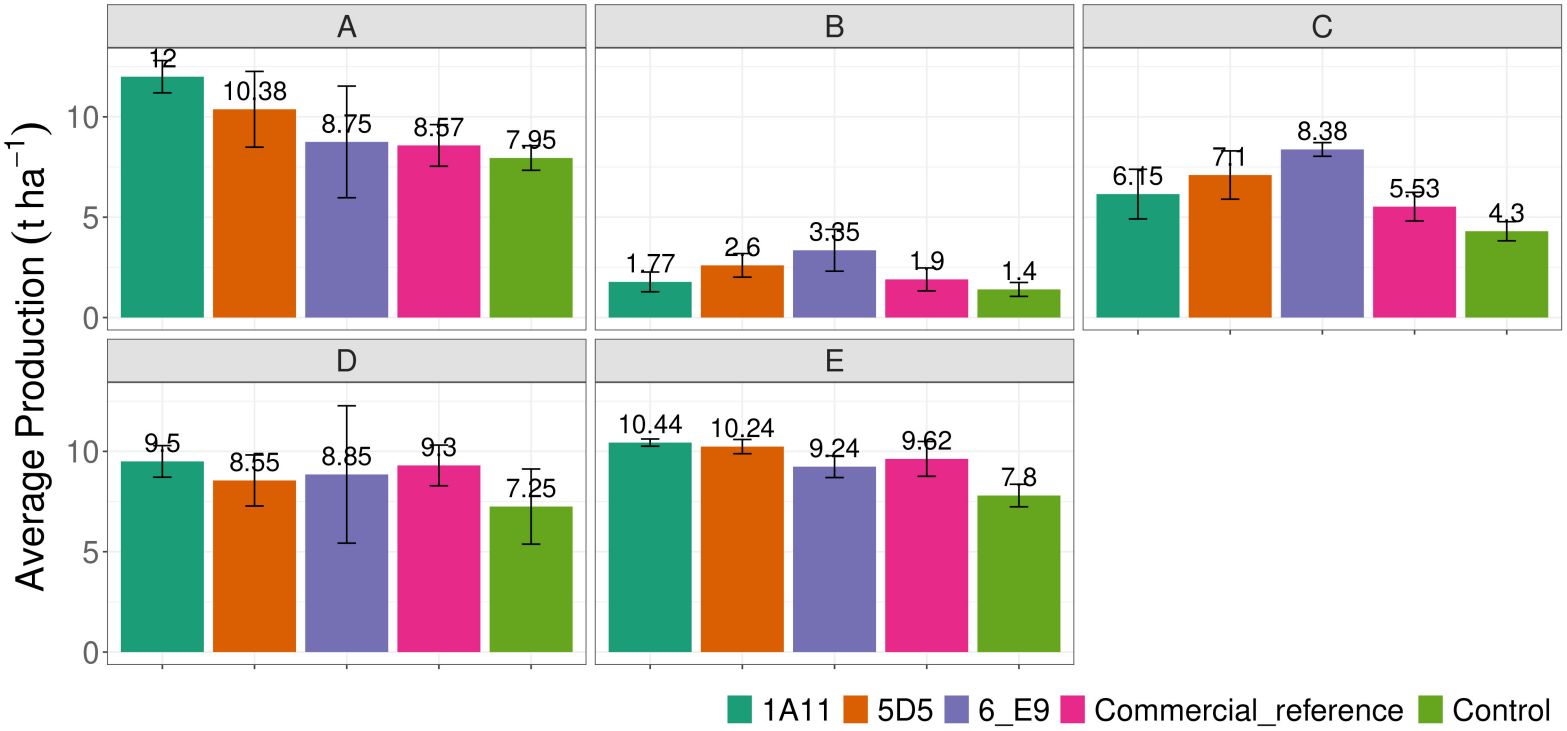
Average productivity (t ha^−1^) of treatments in different locations **(A–E)**. The bars represent the averages and the error bars correspond to the standard deviation of the observations in each treatment within each location. The values above the bars indicate the observed averages.

The descriptive analysis of average productivity (t ha^−1^) indicates variation between the five locations (A-E) and between the treatments evaluated (**Figure 7**). In general, the highest average productivity was observed at locations A, D, and E, while location B showed the lowest. Location C showed intermediate values.

Among the treatments, it is observed that the strain of *B. subtilis* 1A11 and the strains of *B. velezensis* 5D5 tends to show higher productivity in various environments, while the uninoculated control treatment consistently presents the lowest average values. Treatment with the strain of *B. velezensis* 6E9 shows variable performance between locations, with relatively higher values in some environments. Furthermore, variation in data dispersion is observed between treatments and locations, as indicated by the error bars, suggesting differences in variability between experimental environments.

**Figure 8:**
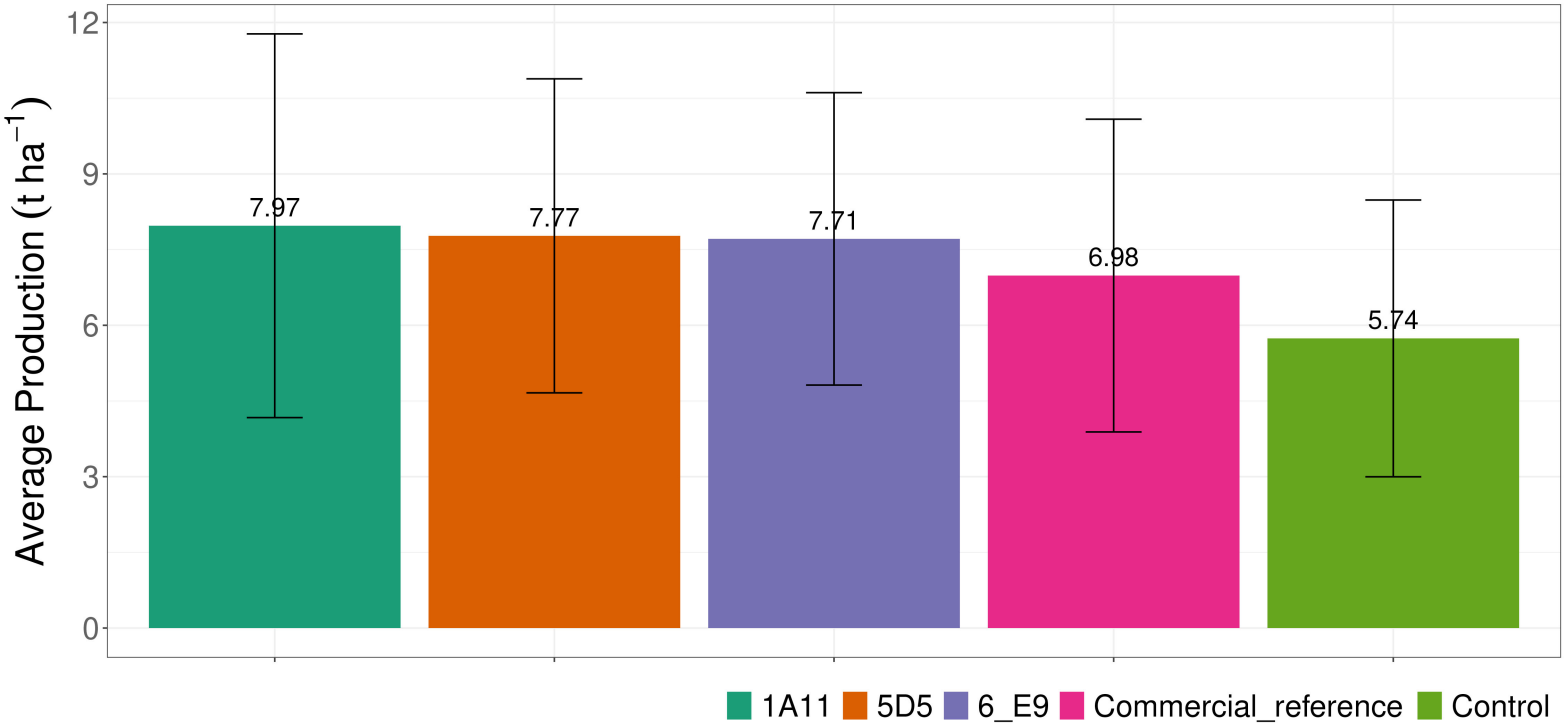
Average productivity (t ha^−1^) of treatments considering all experimental locations together. The bars represent the overall averages and the error bars correspond to the standard deviation of the observations. The values above the bars indicate the observed averages.

In addition, considering all experimental locations together (**Figure 8**), treatments 1A11 (7.97 t ha^−1^), 5D5 (7.77 t ha^−1^), and 6E9 (7.71 t ha^−1^) exhibited the highest overall mean grain yield. These were closely followed by the commercial standard (6.98 t ha^−1^), while the uninoculated control showed the lowest productivity (5.74 t ha^−1^).

Initially, the field data were analyzed using analysis of variance (ANOVA). However, the assumptions of normality and homogeneity of variances were not fully satisfied. The Shapiro-Wilk test (Shapiro and Wilk, 1965) applied to the model residuals indicated a significant deviation from normality (*W* = 0.941, *p <* 0.001). In addition, the Levene test (Levene, 1960), also applied to the model residuals, indicated heterogeneity of variances among locations (*F* = 3.396, *p* = 0.012), although no significant heterogeneity was observed among treatments (*p* = 0.102).

Therefore, the nonparametric Friedman test (Friedman, 1937; Conover, 1999) was chosen because it is appropriate for randomized block designs when the assumptions of analysis of variance are not met.

It should be noted that, although **Figure 7** presents the means for visualization purposes, the Friedman test is based on the ranking of observations within each block, not directly using the mean values. In this procedure, the observations within each block are replaced by their respective ranks, and comparisons between treatments are performed based on the sums of these ranks.

Since the experiment was conducted at different locations, the Friedman test was applied separately for each location, accounting for the design blocks. When significant differences were detected (*p <* 0.05), multiple comparisons between treatments were performed using the sums of ranks, as described by Conover (1999).

Nonparametric analysis was conducted separately for each experimental location, considering the four blocks of the design. Significant differences were observed at locations B, C, and E (*p <* 0.05), with multiple comparisons of rank sums being performed. At locations B and C, treatment 6E9 showed the highest rank sums, indicating superior performance in these environments, while at location E, treatment 1A11 had the highest value. In contrast, at locations A and D, the Friedman test did not detect significant differences among treatments (*p >* 0.05), implying statistically similar performance among treatments in these environments. The rank sums obtained for each treatment and the respective groupings resulting from the multiple comparisons are presented in **Table 5**. These patterns are consistent with the trends observed in **Figure 7**, reinforcing the superior performance of strains such as 1A11 and 6E9 under specific environmental conditions.

**Table 5:** Sum of treatment ranks for productivity in different locations obtained by the Friedman test.

Treatment.	Location A	Location B	Location C	Location D	Location E
1A11	18^a^	8.0^c^	12^bc^	14.0^a^	18.0^a^
5D5	16^a^	15.5^ab^	15^b^	10.5^a^	15.0^ab^
6E9	10^a^	19.0^a^	20^a^	15.0^a^	9.5^bc^
Commercial standard	9^a^	11.0^bc^	9^c^	13.5^a^	13.5^ab^
Uninoculated	7^a^	6.5^c^	4^d^	7.0^a^	4.0^c^

Treatments followed by the same letter within each column do not differ from each other at the 5% level. r = 4 blocks (replications) were considered in each location.

## 4 Discussion

Previous findings of our group demonstrated the ability of *Bacillus* strains isolated from semi-arid soils of the Caatinga biome in Brazil to significantly increase soybean development and grain yield in multi-location field trials in two experimental areas (Vasconcelos et al., 2025). In the present study, we evaluated the performance of a larger set of bacterial strains isolated from the same area after inoculating maize seeds under hydroponic conditions and in field trials. In addition, whole-genome sequencing was employed to elucidate the genetic basis underlying the beneficial traits of five selected strains.

The strategy of isolating drought-tolerant bacterial strains from Caatinga soils by heat shock treatment followed by selection under low water activity and high temperature successfully enriched spore-forming bacteria of the genus *Bacillus*.

Semi-arid ecosystems such as the Brazilian Caatinga serve as natural reservoirs of microorganisms adapted to long-term water stress while retaining plant growth-promoting capacity (Kavamura et al., 2013). These environments are characterized by low and irregular annual rainfall, high solar radiation, elevated evapotranspiration rates, shallow stony soils, and xerophytic vegetation (Franca Rocha et al., 2024). Despite these constraints, they sustain remarkable microbial diversity, which is partly shaped by heterogeneous microhabitats that differ in aeration, organic matter content, nutrient availability, and ion distribution. The ecological filtering imposed by such conditions likely selects microbial taxa with both stress tolerance and functional plasticity (Bonatelli et al., 2021).

The predominance of *Bacillus* spp. among the drought-tolerant isolates is therefore ecologically coherent. Members of this genus are metabolically versatile and can persist for long periods through endospore formation, allowing survival under heat, radiation, desiccation, and nutrient limitation (Nicholson et al., 2000). Upon inoculation, spores germinate and colonize roots via chemotaxis toward plant-derived compounds, activating growth-promoting functions (Beauregard et al., 2013; Etesami et al., 2023). Their resilience also supports formulation stability, as exemplified by commercial phosphate-solubilizing inoculants such as BiomaPhos^®^ in Brazil (de Oliveira-Paiva et al., 2024). The dominance of Firmicutes in desert soils worldwide, including the Gran Desierto de Altar (Ramos-Madrigal et al., 2024), the Colorado Plateau Desert (Osman et al., 2021), and Asian deserts where they represent over 60% of DNA sequences (An et al., 2013), further reinforces the adaptive significance of this group in arid systems.

All selected isolates exhibited multiple plant growth-promoting (PGP) traits *in vitro*, including EPS production, siderophore synthesis, IAA biosynthesis, phosphatase activity, and, in most cases, biofilm formation and nitrogen fixation (**Table 2**). Rather than acting independently, these traits likely operate synergistically to enhance plant performance, especially under water deficit.

EPS and biofilm formation, observed in most strains, are central to rhizosphere hydration and microbial persistence. EPS matrices improve soil aggregation, reduce evaporative loss, and create hydrated microenvironments that buffer roots and bacteria against desiccation (Morcillo and Manzanera, 2021). Similarly, siderophore production enhances iron acquisition under drought conditions, when Fe^3+^ predominantly occurs as poorly soluble hydroxides. By promoting iron mobilization and facilitating redox cycling, siderophores improve microbial fitness while simultaneously supporting plant nutrition (Truong et al., 2024).

Together, these microbial traits likely contributed to the improved morphological and physiological performance observed under PEG-induced osmotic stress conditions. PEG 6000 induces stress by lowering the osmotic potential of the surrounding nutrient solution, thereby restricting water uptake and simulating dehydration-related conditions. Although this approach primarily reproduces the osmotic component of drought and does not capture the full complexity of soil water deficit, it is widely used to investigate plant adaptive responses to reduced water availability under controlled conditions. This low water potential triggers physiological and molecular responses commonly linked to early drought adaptation, including osmotic adjustment, antioxidant activation, altered hormonal signaling, and root morphological remodeling. The increased root surface area observed under PEG treatment in the inoculated treatments likely reflects root plasticity in response to osmotic stress, representing an adaptive mechanism to enhance water foraging under dehydration-related conditions.

In this context, the IAA production by selected strains may provide a mechanistic explanation for the observed alterations in maize root morphology with and without osmotic stress (**Figures 1**, **2**). Enhanced root development in inoculated seedlings exposed to PEG is consistent with auxin-mediated stimulation of root proliferation, a recognized drought-adaptive strategy (Spaepen and Vanderleyden, 2011; de Zélicourt et al., 2022). Fine-root proliferation is closely associated with enhancing resource acquisition and improved water-use efficiency, increasing drought resilience in maize and other cereals (Kost et al., 2026; Lynch, 2022). The higher SPAD index in inoculated plants under stress further suggests preservation of chlorophyll content and photosynthetic capacity, reflecting integrated improvements in nutrient acquisition, hormonal balance, and oxidative stress mitigation (**Figure 1**).

Under stressed and non-stressed conditions, strain 1A11 consistently outperformed the other strains in multiple traits, highlighting its superior performance and robustness and indicating that this strain can be selected for use as a plant inoculant, as it presents benefits under both stress and non-stress conditions (**Figure 1**; **Table 3**).

Principal component analysis integrated these responses. Under osmotic stress, uninoculated controls separated clearly from inoculated treatments (**Figure 2**). Strains 1A11, 2C5, and 2G5 were associated with fine-root traits, total dry weight and chlorophyll content, whereas 5D5, 2E7, 1H10 and 6E9 correlated with increased root diameter and root dry weight, indicating alternative adaptive strategies based on carbon allocation and root thickening. This functional diversification supports the view that drought tolerance emerges from multiple complementary phenotypes rather than a single optimal architecture (Comas et al., 2013; Lynch, 2022). Under non-stressed conditions, strain 1A11 remained strongly associated with root development and increased biomass accumulation (**Figure 2**), indicating broad-spectrum growth promotion. Such stability across environmental contexts strengthens its agronomic value, particularly in regions characterized by high rainfall variability. These patterns indicate that while some strains act as general growth promoters, others show greater benefits under stress conditions.

Genome-based phylogeny identified two species clusters: strains 6E9 and 5D5 aligned with *Bacillus velezensis*, whereas 2E7, 1A11, and 1H10 grouped within the *B. subtilis* clade (**Figure 4**). High-performing strains occurred in both lineages, suggesting that drought resilience and growth promotion depend more on specific genomic configurations than on phylogenetic placement. Comparative genomics revealed conserved genes involved in osmotic adjustment, oxidative stress response, nutrient acquisition (N, P, Fe), motility, chemotaxis, EPS and biofilm biosynthesis, phytohormone production, volatile organic compound synthesis, and general stress responses (see **Supplementary Table 1**).

Antioxidant defense and osmoprotectant accumulation are central drought-adaptive mechanisms (Vaghela et al., 2026). Genomic analysis revealed genes encoding superoxide dismutases (*sodA*, *sodB*, *sodC*, *sodMn*), catalase (CAT), peroxidases (AHP, APX), and pathways for low-molecular-weight antioxidants that support mitigation of reactive oxygen species (ROS) damage and preservation of cellular integrity under waterlimited conditions (Sandhya et al., 2010; Batool et al., 2020). Concurrently, genes involved in compatible solute synthesis and uptake (e.g., *glpF*, *opu*, *betI*, *gbsB*, *cdD*, *tre*) enable accumulation of proline, glycine betaine, choline, and trehalose, stabilizing proteins and membranes under dehydration (Barnawal et al., 2019) (see **Supplementary Table 1**).

All strains harbored genes for tryptophan-dependent auxin biosynthesis (IPA and tryptamine pathways) and the *trpAF* cluster, consistent with their measured IAA production. Although the IAA production levels observed in the strains evaluated here were moderate, maize seedlings inoculated with these bacteria exhibited significant increases in root length and root surface area compared to uninoculated controls (**Figure 3** and **Table 2**). The observed root stimulation likely reflects synergistic interactions among hormonal, nutritional, and protective mechanisms.

Genomic evidence of robust carbohydrate metabolism, including tricarboxylic acid, Entner–Doudoroff, Embden–Meyerhof–Parnas, and pentose phosphate pathways, supports efficient utilization of root exudates (Chagas et al., 2018; Ray et al., 2018), facilitating rhizosphere colonization. Motility (*fli*, *flg*, *mot* operons) and adhesion systems such as EPS and biofilm-related genes (*eps* and *glg* operons) further enhance colonization potential, thereby strengthening root–soil interactions (Bogino et al., 2013; Vlamakis et al., 2013; Deng et al., 2015; Saha et al., 2020). Exopolysaccharides (EPS), composed of hydrated polymers, play a central role in enhancing plant growth and survival under drought conditions by protecting roots against desiccation and improving soil water retention. Through biofilm formation, EPS-producing bacteria strengthen plant-microbe interactions, facilitate nutrient exchange and signaling, and protect roots from pathogen attack. Additionally, these biofilms create a microenvironment with higher moisture and nutrient availability, enhancing root hydraulic conductivity, improving soil structure, and increasing plant resilience under severe water deficit.

Nutrient acquisition traits substantially contributed to the functional performance of the strains. Most isolates exhibited *in vitro* nitrogen fixation capacity, while the presence of conserved *pst*/*pho* operons and related phosphate transport genes supports efficient phosphorus uptake and solubilization. Considering the diffusion limitations of phosphorus in dry soils, microbial-mediated mobilization may alleviate nutrient stress and sustain plant energy metabolism under water deficit. In addition, iron acquisition systems (*suf*, *fhu*, *ent*, ABC transporters and associated genes) further enhance plant–microbe resilience by improving chlorophyll synthesis and antioxidant capacity while potentially suppressing phytopathogens. The presence of operons related to the methylcitrate cycle, propionyl-CoA metabolism, glycerate metabolism, and *α*acetolactate pathways further indicates a metabolic capacity for organic acid production, a key mechanism for mineral chelation and enhanced solubilization of phosphorus and micronutrients such as iron and zinc.

Effective validation of a novel technology requires assessment under conditions that closely replicate realworld applications. In agricultural systems, it is important to account for both predictable and unpredictable environmental variability, including excess or lack of rainfall, thermal stress outside optimal ranges, edaphoclimatic variability, and biotic pressures such as endemic diseases and pests. Only after evaluation under realistic and heterogeneous conditions can the true potential of a technology be properly determined. Thus, field trials were conducted under realistic cultivation conditions across five different locations during the 2024 season and were associated with improved performance under water-limited conditions (**Figure 6**). Under non-irrigated field conditions, with a declining rainy season (**Figure 6**), the strain 1A11 exhibited mean productivity values superior to those of the uninoculated control across all experimental fields. In comparison to the commercial standard, strain 1A11 outperformed it in Locations A, C, and E, exhibited comparable performance in Location D, and was inferior in Location B (**Figure 7**).

However, it is important to note that Location B experienced the lowest cumulative rainfall (250.9 mm), along with irregular rainfall distribution and repeated episodes of temperatures exceeding 40 °C during the cropping period (**Figure 6**). These environmental conditions were severely limiting for maintaining commercially viable grain production, resulting in average yields ranging from 1.4 t ha^−1^ to 3.35 t ha^−1^ across all treatments (**Figure 7**).

Strain 5D5 also resulted in average productivity above that of the uninoculated control and surpassed the reference inoculant in four locations (A, B, C, and E), with lower performance than the commercial standard, solely in Location D. Similarly, strain 6E9 consistently outperformed the uninoculated control across all locations, and exhibited higher mean productivity than the commercial standard only in Locations B and C, while demonstrating comparable performance in Locations A, D, and E. These findings indicate that the *Bacillus* strains 1A11, 5D5, and 6E9, as well as the commercial bioinoculant used as reference, effectively enhanced maize productivity during the summer-to-autumn transition in Brazil, a period corresponding to the second maize crop season, which is typically characterized by conditions of water deficit throughout the crop cycle in rainfed fields (**Figure 8**).

**Table 1** shows the variability in the physicochemical characteristics of the soils across the experimental fields evaluated in this study. When these data are interpreted alongside the climatic conditions observed during the crop cycle, the likely influence of soil characteristics on moisture availability and crop performance becomes evident. Although no soil moisture measurements were taken during the experiment, it is known that soils with a predominantly sandy composition and low organic matter have a lower capacity to retain water in the soil profile for longer periods than clayey soils with more organic matter. For instance, although locations B and E experienced similarly restrictive rainfall regimes, the reduction in yield was considerably greater in location B, characterized by sandy soil with lower fertility and reduced organic matter content, compared with location E, which had clayey soil with higher fertility. These conditions are characteristic of intermittent drought stress (“veranicos”), which frequently occur in Brazilian rainfed systems and substantially affect maize productivity cultivated during the second crop season (“safrinha”) under non-irrigated conditions. Therefore, the severe reduction in grain yield observed in Location B strongly supports the occurrence of environmentally restrictive conditions during the crop cycle. These differences likely contributed to improved soil water retention and nutrient supply in location E, thereby supporting better plant development and higher productivity under water-limited conditions.

An expected finding was the substantial variation in productivity levels across locations, independent of treatment effects. This heterogeneity can be attributed to the pronounced environmental differences observed among sites over the course of the experiment, primarily driven by variation in rainfall, but also by differences in soil texture and fertility among the experimental locations. Nevertheless, despite these contrasting edaphoclimatic conditions, the evaluated *Bacillus* strains 1A11, 5D5 and 6E9, consistently enhanced crop productivity compared with both the uninoculated control and the commercial inoculant treatment. Drought stress in agricultural systems is a multifactorial phenomenon strongly modulated by soil water-holding capacity and edaphic properties, not exclusively by cumulative rainfall (Wijitkosum, 2025), as the results obtained in this study indicate. Cumulative precipitation ranged from 250.9 mm at location B to 599.8 mm at location D (**Figure 6**), exposing maize plants to different soil moisture levels throughout the growing cycle. In a related study, Vasconcelos et al. (2025) reported similar location-dependent fluctuations in soybean productivity when evaluating the same *Bacillus* strains, which they also attributed to environmental variability, particularly due to differences in rainfall.

Importantly, the superior performance of Caatinga-derived strains highlights the advantage of selecting inoculants from environments shaped by comparable selective pressures, thereby supporting ecological matching as an effective strategy to improve crop resilience. Although the selected Bacillus strains promoted maize performance under field conditions, the mechanisms underlying their establishment and interaction with native microbial communities remain to be elucidated. Microbial inoculation can modify rhizosphere community composition through direct microbial competition, production of antimicrobial compounds, niche occupation, biofilm formation, or indirect plant-mediated mechanisms such as altered root exudation patterns (Bakker et al., 2013; Compant et al., 2019). Depending on ecological compatibility, inoculated strains may establish synergistic interactions with resident microbiota or experience antagonistic exclusion, ultimately influencing inoculant persistence and efficacy (Kaminsky et al., 2019). Furthermore, environmental stress conditions, including drought, may intensify microbial selection processes and reshape microbial network dynamics in the rhizosphere (Naylor and Coleman-Derr, 2018). In the present study, although microbial community composition was not assessed, the positive field responses suggest that the selected strains were capable of functioning within the native microbial environment. Future studies integrating amplicon sequencing, metagenomics, and strain-tracking approaches are warranted to investigate microbial succession, persistence, and ecological interactions after inoculation.

Consistent with their demonstrated efficacy, the strain of *B. subtilis* 1A11, and the strains of *B. velezensis* 5D5 and 6E9 were registered with the Brazilian Ministry of Agriculture, Livestock and Food Supply (MAPA) as commercial inoculants for soybean and maize. This achievement resulted from a collaborative partnership between Brazilian Agricultural Research Corporation (Embrapa) and the inoculant companies Bioma and Simbisose, integrating scientific research with industrial development to translate experimental findings into applied innovation. In May 2025, this collaboration culminated in the commercial launch of Hydratus^®^, formulated with *B. subtilis* strain 1A11, marking a significant step toward the adoption of climate-resilient microbial technologies in Brazilian agriculture.

## 5 Conclusion

The *Bacillus* strains evaluated in this study exhibited multiple plant growth-promoting traits related to drought tolerance, including EPS and biofilm production, nutrient acquisition, and the ability to grow under low-water conditions. Several strains effectively modulated root system morphology in early maize development, indicating the potential to enhance tolerance to abiotic stress. Among them, strains 1A11, 5D5, and 6E9 converted these traits into consistent agronomic gains, significantly increasing grain yield under rainfed field conditions across multiple environments.

Genomic and phenotypic evidence revealed a coordinated, multifunctional strategy involving osmoprotection, antioxidant defense, phytohormone production, improved nutrient availability, and EPS/biofilm formation indicating synergistic mechanisms that enhance plant performance under waterlimited conditions. The superior and stable field performance of the evaluated strains highlights their potential as effective microbial biostimulants for sustainable agriculture. Moreover, the strains maintained positive agronomic effects across contrasting edaphoclimatic conditions, demonstrating robustness under diverse environmental scenarios. Therefore, field performance conclusions are not based solely on precipitation data, but rather on the integrated interaction among climatic conditions, soil characteristics, and the effects of microbial inoculation.

Overall, these findings highlight the potential of Caatinga-derived *Bacillus* strains as climate-resilient technologies, demonstrating both field applicability and successful development into commercial bioinoculants.

## Data Availability

The datasets presented in this study can be found in online repositories. The names of the repository/repositories and accession number(s) can be found in the article/**Supplementary Material**.
